# Spatio-Temporal Magnitude and Direction of Highly Pathogenic Avian Influenza (H5N1) Outbreaks in Bangladesh

**DOI:** 10.1371/journal.pone.0024324

**Published:** 2011-09-09

**Authors:** Syed S. U. Ahmed, Annette K. Ersbøll, Paritosh K. Biswas, Jens P. Christensen, Nils Toft

**Affiliations:** 1 Department of Large Animal Sciences, Faculty of Life Sciences, University of Copenhagen, Frederiksberg, Copenhagen, Denmark; 2 Department of Medicine and Surgery, Faculty of Veterinary Medicine, Chittagong Veterinary and Animal Sciences University, Chittagong, Bangladesh; National Institute of Public Health, University of Southern Denmark, Copenhagen, Denmark; 4 Department of Microbiology, Faculty of Veterinary Medicine, Chittagong Veterinary and Animal Sciences University, Chittagong, Bangladesh; 5 Department of Veterinary Disease Biology, Faculty of Life Sciences, University of Copenhagen, Frederiksberg, Copenhagen, Denmark; University of Hong Kong, Hong Kong

## Abstract

**Background:**

The number of outbreaks of HPAI-H5N1 reported by Bangladesh from 2007 through 2011 placed the country among the highest reported numbers worldwide. However, so far, the understanding of the epidemic progression, direction, intensity, persistence and risk variation of HPAI-H5N1 outbreaks over space and time in Bangladesh remains limited.

**Methodology/Principal Findings:**

To determine the magnitude and spatial pattern of the highly pathogenic avian influenza A subtype H5N1 virus outbreaks over space and time in poultry from 2007 to 2009 in Bangladesh, we applied descriptive and analytical spatial statistics. Temporal distribution of the outbreaks revealed three independent waves of outbreaks that were clustered during winter and spring. The descriptive analyses revealed that the magnitude of the second wave was the highest as compared to the first and third waves. Exploratory mapping of the infected flocks revealed that the highest intensity and magnitude of the outbreaks was systematic and persistent in an oblique line that connects south-east to north-west through the central part of the country. The line follows the Brahmaputra-Meghna river system, the junction between Central Asian and East Asian flyways, and the major poultry trading route in Bangladesh. Moreover, several important migratory bird areas were identified along the line. Geostatistical analysis revealed significant latitudinal directions of outbreak progressions that have similarity to the detected line of intensity and magnitude.

**Conclusion/Significance:**

The line of magnitude and direction indicate the necessity of mobilizing maximum resources on this line to strengthen the existing surveillance.

## Introduction

Highly pathogenic avian influenza A subtype H5N1 virus (hereafter HPAI-H5N1) is a zoonotic virus primarily affecting poultry, but with a high case fatality rate in infected humans. Since 2003, more than 60 countries have registered the virus in their territories [1]. Bangladesh officially reported its first HPAI-H5N1 outbreak on 22 March, 2007 [2]. By March 2011, a total of 480 outbreaks had been reported in 49 of the 64 districts that placed the country among the highest reported numbers worldwide [1]. So far, the country confirmed three human cases without fatality [3]. More than 1.8 million chickens were culled, and because of HPAI-H5N1 outbreaks an economic loss of US$ 40 million was estimated for the year 2008 alone [4]. Therefore, prevention and control of HPAI-H5N1 has a high priority in Bangladesh. Despite the endeavors to explore associated risk factors [2], [5], [6] and space-time clusters [6], [7], the understanding of the epidemic progression, direction, intensity, persistence and risk variation over space and time remains limited. A better understanding of the epidemic behavior could help the country for future surveillance and control program.

Bangladesh is a resource-limited country and needs an epidemiological knowledge-based approach to target the control and potentially eradicate the disease. Bangladesh does not have sufficient resources, including veterinary services, to conduct nationwide active surveillance for HPAI-H5N1. Bangladesh could, however, potentially utilize the mandatory disease reporting system of the World Animal Health Organization (OIE), and readily available sources of geographical risk factors like FAO geo-network (http://www.fao.org/geonetwork) for defining high risk areas and get more insight to the disease outbreaks. Hence, the country could define high risk areas based on reported information and available geographical risk factors and target those areas to intensify the surveillance and control effort.

Inference on large-scale infectious disease epidemics in animal populations can be assessed based on synthesis of information accumulated from a variety of sources. Information could potentially include field observations, details provided by those with local knowledge of the area of investigation, surveillance data of outbreak, geographical data and demographic data [8]. Geographic Information Systems (GIS) have the ability to handle and combine such a variety of data sources and is, therefore, being used increasingly to study the spatial and temporal patterns of infectious diseases [9]. The application of GIS and geostatistics help to gain additional insight into the introduction, spread and persistence of the transboundary animal diseases like HPAI-H5N1 by utilizing the information from the mandatory reporting systems. This could potentially help advocating an enhanced control program and the design of an empirical surveillance system [10], [11].

The available epidemiological data from the mandatory disease report system on the different waves of HPAI-H5N1 outbreaks in Bangladesh, and geographical risk factors have provided an opportunity to explore the magnitudes of the outbreaks over space and time. In the current study, we concurrently used descriptive exploratory methods and geostatistical analyses to depict the spatial and temporal components of the outbreaks in order to gain an in-depth understanding of the introduction, spread and persistence of the HPAI-H5N1 outbreaks in Bangladesh from the available data.

## Materials and Methods

### Data and data managements

For the present study, we included data for 325 HPAI-H5N1 flock outbreaks in Bangladesh that occurred from February 2007 through December 2009, officially reported to the World Animal Health Organization (OIE) by the Department of the Livestock services (DLS), Bangladesh [1]. For each official outbreak, the virus was confirmed in submitted tracheal samples to the National Reference Laboratory for Avian Influenza (NRL-AI) by the use of reverse-transcriptase polymerase chain reaction (RT-PCR) (7). The official report included physical location, detection date, number of susceptible animals and number of dead and culled birds, as well as geographical coordinates of the poultry farms/holdings. We obtained poultry density data from Food and Agricultural Organization (FAO) via the Geonetwork. These data were available in raster format with pixel value containing estimated flock densities (flock/km^2^). Details on data source and estimation methods were described in the FAO's Gridded Livestock of the World [12]. We obtained flock density data on subdistrict level by using zonal statistical calculation techniques from the global raster data. Data on geographical locations of important migratory bird areas were obtained from the literature [13]–[15].

Directional statistics was focused on subdistrict level of Bangladesh, to portray nationwide outbreaks progression and hence we excluded modeling of flock level local spread. For directional statistics, the location used for the outbreaks at subdistrict level was the centroid of the particular subdistrict and date of an outbreak was the date of the first reported outbreak(s) in that particular subdistrict in the respective wave of outbreaks. If multiple outbreaks took place at the same time and subdistrict, the location and date would be essentially the same; thus we considered it as a single outbreak at subdistrict level. Bangladesh is administratively divided into 481 subdistricts (mean area, 314 km^2^; median area, 271 km^2^; range, 36–1968 km^2^) with variable number of poultry flocks per subdistrict ranging from very small to large populations (mean, 15367; median, 11283; range, 252–254014). Coordinates for the centroids were calculated from the subdistrict level digital map in ArcMap 9.2 (Environmental System Research Institute, Redlands, CA, USA).

We performed data management in Excel (Microsoft, Redmond, WA, USA) and Statistical Analysis System (SAS) version 9.1 (SAS Institute Inc., Cary, NC, USA).

### Exploratory descriptive analysis

To describe the epidemic progression during the study period, we constructed an epidemic curve by plotting, simultaneously, the weekly sum of the number of infected flocks in the primary axis and weekly sum of the infected subdistricts in the secondary axis as a function of time. We performed descriptive statistics by means of frequency distributions (N, %) of the number of spatial units and flocks affected, longevity of the waves, outbreak doubling time, mortality rate before culling, number of birds culled, types of production system affected in three different waves of outbreaks to show the behavior and magnitude of the epidemic waves.

We performed exploratory descriptive analysis in Excel (Microsoft, Redmond, WA, USA) and Statistical Analysis System (SAS) version 9.1 (SAS Institute Inc., Cary, NC, USA).

### Exploratory spatial analysis

Exploratory mapping was used to describe the geographical distribution of the infected flocks, poultry flock density and important migratory bird areas.

We used kernel density estimation (KDE) to obtain a smoothed map of the outbreak pattern of the different epidemic waves from infected flock locations. KDE is used to estimate the first order properties of a spatial stochastic process describing the global or large scale trend and can be shown in terms of the intensity of the process, which is the mean number of events per unit area of the points in a specific region [16]. A 10 km bandwidth of the kernel was used. This was arbitrarily chosen as it maintained the balance between detail and smoothness in exploring the first order properties of the outbreak pattern [17].

Empirical Bayes (EB) flock level incidences were calculated for the individual subdistricts during the three different waves of outbreaks. Based on the EB, maps describing the spatial variation in risk of outbreaks taking into account the distribution of the population poultry flock at risk at subdistrict level were generated. The EB technique adjusted the incidence rate, especially in areas with a small population estimates by a weighted compromise between the crude rate, the overall mean relative rate, and a local mean of the relative rate in nearby areas [18].

The exploratory mapping and ordinary KDE was performed using the spatial analyst tool of ArcGIS 9.2 (Environmental System Research Institute, Redlands, CA, USA). EB was calculated in GeoDa 0.9.5-I (Arizona State University, USA) and mapped in ArcGIS 9.2.

### Geostatistical analysis

We used a directional statistics to test the null hypothesis that no association exists between the times at which subdistricts were infected and the overall directions of the vectors formed by connecting the spatial locations of the subdistricts became infected over the outbreaks wave [19]. Briefly, a chain of the individual infected subdistricts was constructed in ascending order of the outbreak date (the earliest case first, then the second case and so on). A line was then drawn to connect the location of the first subdistrict faced the outbreak to the second subdistrict and continued until all the subdistrict locations were connected to form a time-connection-matrix. The chain had at least two ends (the first and last cases), also branches if multiple cases took place at the same time. We examined the chain of cases considering if they were relative (each outbreak is related to all outbreaks that followed it). The test statistic is a vector that includes both direction and magnitude. The direction of the vector points in the average direction of advance of the spread of outbreaks. We can describe the direction of any vector in terms of its counterclockwise angle of rotation from horizontal, with East corresponding to 0°. The magnitude is the angular variance of these links. When the links point in the same direction, the angular variance is small, and when they point in many directions the angular variance is large. Thus, the angular variance, also termed angular concentration, describes the consistency of spreads and is in the range of 0 to 1. The significance of the test statistic was obtained using 999 Monte Carlo simulations in ClusterSeer version 2.0 (TerraSeer Inc. 2002, Ann Arbor, MI, USA).

## Results

### Exploratory descriptive analysis

From February 2007 through December 2009, a total of 325 outbreaks with laboratory confirmation of HPAI-H5N1 were officially recorded. During this period, 148 (31%) of the 481 subdistricts had officially confirmed HPAI-H5N1 outbreak(s); there were 50 subdistricts with >1 officially confirmed outbreaks.

**Figure 1:**
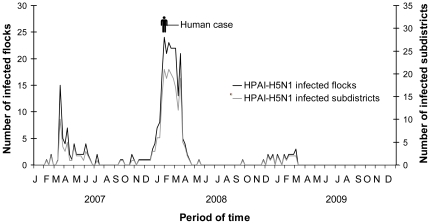
Weekly number of HPAI-H5N1 infected flocks and subdistricts, Bangladesh, 2007–2009.

The epidemic curves of HPAI-H5N1 infected flocks and subdistricts per week are shown in Figure 1. The black curve indicates the flock level magnitude and the grey curve gives an indication of the spatial extent (infected subdistricts) of the outbreaks. These two curves show an epidemic pattern of HPAI-H5N1 outbreaks with three independent waves. The first wave occurred from February 2007 through July 2007 with a peak in March 2007; the second wave from December 2007 through April 2008 with the peak in February 2008; and the third, less distinct wave from December 2008 through March 2009 with no detectable peak. In Table 1, a summary of the descriptive analysis regarding the longevity, magnitude and spatial distribution of the different waves are given.

**Table 1:** Summary of the longevity, magnitude, spatial distribution and mortality of the three different waves of HPAI-H5N1 outbreaks in Bangladesh, 2007–2009.

Indicator	First wave	Second wave	Third wave
Number of infected districts (n = 64)	20	45	14
Number of infected subdistricts (n = 481)	42	112	22
Number of infected flocks	68	225	32
Length of the wave (days)	132	131	122
Outbreaks doubling time (days)*	21.68	16.77	24.40
Backyard (% of total infected flocks)	38.2	7.5	33.3
Commercial (% of total infected flocks)	61.8	92.5	66.7
Mortality rate before culling (%)	14.1	18.9	7.9
Number of dead birds	43826	231950	3458
Number of culled birds	267545	998864	41917
Human case (Case/Death)	0/0	1/0	0/0

*Formula used for the calculation of outbreaks doubling time is given in Text S1.

### Exploratory spatial analysis

**Figure 2:**
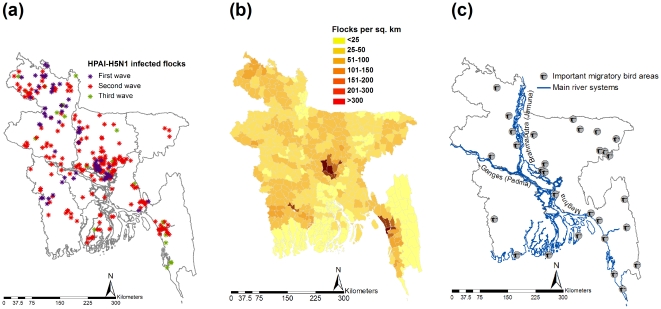
Spatial distribution of (a) HPAI-H5N1 infected flocks in poultry from February 2007 through December 2009; (b) poultry flock density in Bangladesh; (c) known migratory bird winter stopovers in Bangladesh.

The distribution of the HPAI-H5N1 outbreaks in poultry from February 2007 through December 2009, the poultry density in Bangladesh and the important migratory bird areas in Bangladesh are shown in Figures 2a, 2b and 2c, respectively.

**Figure 3:**
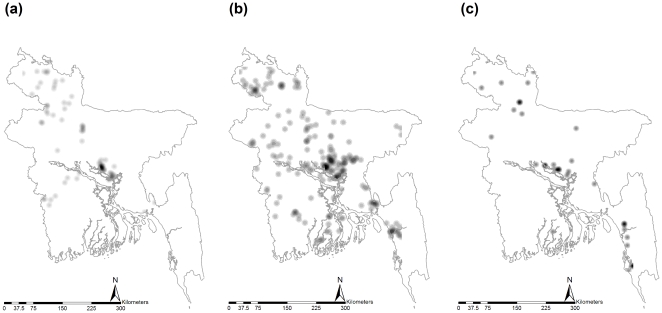
Kernel density estimation of spatial distribution of HPAI-H5N1 in poultry flocks in Bangladesh from 2007 through 2009: (a) first wave (b) second wave (c) third wave. A darker area corresponds to higher density.

Kernel density estimation maps show the first order spatial property of the HPAI-H5N1 outbreaks by the waves (Figures 3a, 3b and 3c). The estimated maps portray that the geographical location of highest intensity and magnitude of outbreaks in the three different waves were similar but with little variation. Overall, outbreaks locations were in an oblique line that connects south-east to north-west through the central part of the country.

**Figure 4:**
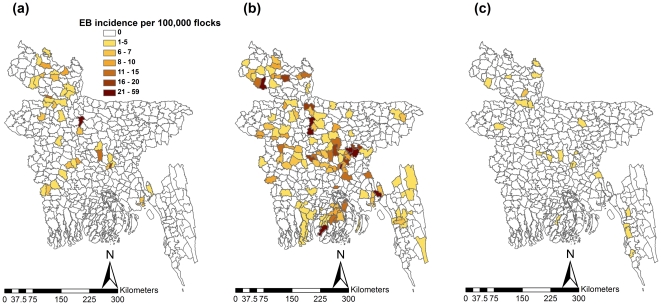
Emperical Bayes flock level incidence of the HPAI-H5N1 outbreaks, by subdistricts, for three epidemic waves during 2007–2009. (a) First wave. (b) Second wave. (c) Third wave (legend category is the same for all maps).

The EB incidence rate for describing the variation of incidence of HPAI-H5N1 outbreaks at subdistrict level of Bangladesh for the three different waves is presented in Figures 4a, 4b and 4c. The mapping substantiated the results of kernel smoothing and confirmed that the subdistricts with higher risk are located along an obliquely straight line connecting north-western to south-eastern parts at the ends through the central parts in the middle.

### Geostatistical analysis

Results of the directional statistics for three different waves are presented in Table 2. In the first and second wave of outbreaks, we observed a significant (p = 0.001) overall outbreak direction. In the third wave, overall outbreak direction was borderline insignificant (p = 0.058). Assuming that the outbreak time connection matrix is relative the average angle of the spread for fist wave was 131° (south-east to north-west), for the second wave was 317° (north-west to south-east) and for third wave was 283° (north-west to south-east).

**Table 2:** Results of directional statistics for three different waves of HPAI-H5N1 outbreaks in Bangladesh, 2007–2009.

Wave	Average angle	Angular concentration	P	Direction
One	131	0.391	0.001	NW-SE
Two	317	0.075	0.001	SE-NW
Three	283	0.061	0.059	SE-NW

## Discussion

The distribution of HPAI-H5N1 outbreaks of the three waves in Bangladesh was clustered in time, occurring during winter and spring. That suggests a possible role of migratory birds for introduction and subsequent spread of the virus during their latitudinal (north to south or vice versa) migration in winter and spring [20] following the Central Asian Flyway and East Asian Flyway over the Bangladesh [21]. A comparison between the three different waves shows that the magnitude including reported number of the outbreaks were highest in the second wave. Outbreak doubling time of wave two was smaller than other waves (Table 1) indicating rapid spread of outbreaks than the two other waves. A possible explanation is that in the second wave outbreaks mostly took place in the commercial poultry production circuits. In the commercial production system, the contact rate is much higher than in backyard system that favors rapid transmission and spread among the susceptible population. The number of outbreaks markedly dropped in the third wave. However, HPAI-H5N1 seems to persist within Bangladesh causing intermittent sporadic outbreaks also in the year 2010 [22].

Exploratory mapping of the geographical distribution of infected flocks (Figure 2a), first order trends of the distribution of infected subdistricts (Figures 3a, 3b and 3c) and flock level EB-incidence of subdistricts (Figures 4a, 4b and 4c), portray that the highest intensity and magnitude of outbreaks in the first wave was in the central and north-western parts. In the second wave, the highest magnitude remained in the same two geographical locations with an addition of a south-eastern part, and the highest intensity of outbreaks in the third wave was also reported from the same three parts: north-western, central and south-eastern. Based on these results the central part of the country can be considered as the highest risk area followed by north-western and south-eastern part. This is in accordance with an earlier study that identified significant clusters of HPAI-H5N1 infected subdistricts in different production systems, in the same locations of Bangladesh [7]. The geo-spatiality of the high risk areas is persistent in an oblique line that connects south-east to north-west through the central part of the country (hereafter referred to as the ‘hot spatial line’). The hot spatial line indicates that there is a nonrandom pattern of the HPAI-H5N1 outbreaks in Bangladesh and some common mechanism of introduction, spread and persistence, suggesting the possibility of formation of a niche of the virus. This niche could play roles in spillover and spillback of the virus in the transmission cycle making HPAI-H5N1 epidemiology more complicated.

Three factors likely contributed to the genesis and persistency of hot spatial line of the HPAI-H5N1 outbreaks in Bangladesh. First, several important migratory bird areas (Figure 2b) have been identified along the Brahmaputra-Meghan river system (Figure 2c) that coincides with the hot spatial line. The river systems play an important role as key staging areas for migratory birds during their migration [23]. This finding might generate a hypothesis that migratory birds could be an important actor for the genesis of the hot spatial line by the introduction and subsequent spread of HPAI-H5N1 every year in Bangladesh. Satellite-marked tracking of waterfowls revealed that different ducks and gulls species, which faced high mortality during the HPAI-H5N1 outbreaks in Qinghai Lake in 2005 [24]–[26], can migrate from China and in particular from Qinghai Lake to Bangladesh following different routes [15], [27]. A recent work based on satellite telemetry confirms that the location and time of migration of wild migrant birds from China and Mongolia coincides with HPAI-H5N1 in Bangladesh [28]. Second, poultry production is generally extensive along the Central Asian Flyways [29]. The poultry population density is much higher along the hot spatial line (Figure 2b). For maintaining an infectious chain and persistence of HPAI-H5N1, it requires high density and sustained host population, which increases the contact and transmission rate [30], [31]. Third, the highway connecting the south-east to the north-west through the central part of the country is the main poultry trading route in the country [32]. Transportation of infected poultry, contaminated poultry product and equipment through this route may be playing a role. Previous studies confirmed the role of the highway network in HPAI-H5N1 epidemiology of different countries [10], [33], [34]. Further studies of the role of trading routes and markets are needed.

The findings of the directional analyses of the three different waves suggest that the direction of outbreaks was latitudinal (north to south or vice versa). The latitudinal migration of wild birds takes place in Bangladesh following Central Asian Flyway and East Asian Flyway [21]. Both, the northward and southward migration, can be observed by different species [20]. Different ducks and gulls species, which faced high mortality during the HPAI-H5N1 outbreaks in Qinghai Lake in 2005 [24], [25], can migrate from China and Mongolia to Bangladesh following different routes [15], [27]. The result of the first wave indicates that the outbreaks were resulted from northward migration of some species. This has similarities with the described route and time of the flight of the migratory birds in an earlier study [15]. Gull species marked at Qinghai Lake of China, tracked for their movements, reached the south-eastern and southern part of Bangladesh in December to January, and some of them moved to the central part of Bangladesh during March [15]. A more recent work based on satellite telemetry, corroborates our finding, which summarized the spreading pattern of HPAI-H5N1 from 2005 through 2009 in South Asia in a direction from southern to the northern part of the continent following the Central Asian Fly ways [28]. The direction of the second and third wave was northward, indicates the importance of latitudinal migration. This finding might generate a hypothesis that migratory birds could reintroduce the virus every year to initiate a new wave of outbreaks and subsequent spread. The hypothesis of reintroduction of the same lineage H5N1 virus into Bangladesh every year by the migratory birds is challenged by (1) the fact that sporadic incidences were also reported well after the wild bird migration period and (2) phylogenetic analysis of the virus. Phylogenetic analysis on the virus isolates of Bangladesh between 2007 and 2009 revealed that they all belonged to the subclade 2.2 of the Qinghai lineage and genetically were very close except some minor mutation [6], [35]. However, the possibility of reintroduction of the same virus by migratory birds cannot be resolved until persistence of the virus in them has been thoroughly demonstrated.

The inferences drawn from this study is based on the assumption that almost all the outbreaks of HPAI-H5N1 occurred in Bangladesh during the study period were reported. In the current surveillance system, detection of HPAI firmly depends on the clinical signs of the disease including high mortality in the flock. Due to the high mortality rate of HPAI and a compensation plan of the government in place the risk of underreporting of clinical cases is considered negligible. However, random underreporting of the outbreaks and inaccuracy in outbreak date is possible. Random underreporting of flock level outbreaks would cause underestimation of the intensity of the outbreaks and of the Empirical Bayes incidence, suggesting even stronger intensity and estimate. Random inaccuracy of the outbreak date will not change the spatial location but might change the time connection slightly. In the directional statistics, the average direction and its significance is evaluated through a randomization procedure which holds the locations (sine and cosine matrices) constant and randomly assigns connections between pairs of cases by randomizing their times of occurrence. This procedure is also repeated to generate a distribution of the angular concentration under the null hypothesis [19]. Hence, the effect of random inaccuracy in the outbreak dates on results obtained in the directional statistics is considered negligible. Besides, ducks have been demonstrated to be infected without showing clinical signs. This could complicate the epidemiology further.

We assumed that data on all outbreaks were included in this study, and that eventual missing data were missing at random. However, systematic or non-random underreporting cannot be excluded. Such underreporting might be due to human behavior, administrative factors and other ecological factors. If the underreporting has some spatial trend, it may potentially bias the findings in this study and increase the uncertainty of the estimates. It is generally not possible to assess the magnitude and consequences of such underreporting without supplementary studies that go beyond the scope of studies such as the present. Thus, we suggest the results are interpreted while keeping the caveats of systematic underreporting in mind.

Like most east and south-east Asian countries, HPAI-H5N1 outbreaks show a seasonality in Bangladesh. HPAI-H5N1 outbreaks in poultry do appear to be most frequent between December and March [36]. However, Thailand exhibited a higher number of outbreaks in July to September and moderate activity in January to February [36]. Like Thailand, in Bangladesh the highest number of outbreaks was also in the second wave. A significant increase in number of outbreaks in Thailand, in the second wave, was probably due to the introduction of a nationwide active surveillance (X-ray survey) [37]. In contrast, the surveillance in Bangladesh was unchanged, and the increase in outbreaks in the second wave, therefore, indicates a different behavior and mechanism of the epidemic. We observed a difference in the spatial risk variation between Bangladesh and some other south-east Asian countries. In Bangladesh, high risk persisted along a well-demarcated spatial line connecting the north-west through the central part to the south-east over all outbreak waves. Such demarcated geographical areas of high risk were not previously observed anywhere. The risk varied between regions over the HPAI-H5N1 outbreak waves in Thailand [37], [38] and Vietnam [39]. These country specific differences indicate that Bangladesh might have a different profile of HPAI-H5N1 outbreaks. Study of the outbreak direction can provide valuable insight on introduction and subsequent spread of outbreaks. Thus, we studied the direction of the HPAI-H5N1 outbreak in Bangladesh. None of the Asian countries studied direction of HPAI-H5N1 outbreak before. Only, the direction of the HPAI-H5N1 outbreak in Romania was reported before [11].

In this paper, we systematically applied exploratory and analytical geospatial statistics to describe the magnitude and spatial behavior of the HPAI-H5N1 outbreaks in poultry from 2007 to 2009 in Bangladesh. This study unravels at least two spatial factors that might have had some roles in initiating new waves of outbreaks, and the perpetuation of the virus in Bangladesh. The factors are: (a) a well-demarcated spatial line connecting the north-west through the central part to south-east of the country having the highest intensities of the outbreaks and (b) important migratory bird areas along the Brahmaputra-Meghna river basin, intensified poultry production and major poultry trading routes near or overlapping the spatial distribution with the highest magnitude of HPAI-H5N1 outbreaks. Such findings are hardly possible in a classical epidemiological setup. Use of GIS and spatial analyses, therefore, provided valuable information concerning the introduction, persistence and spread of the outbreaks in Bangladesh.

Bangladesh depends predominantly on passive surveillance to locate probable HPAI-H5N1 outbreaks based on chicken mortality reported to the DLS local offices by farmers/smallholders [7]. Moreover, scarce resources directed towards eradicating the virus, insufficient epidemiological knowledge on geo-spatial trends of the outbreaks, and the virus entrenchment sources might bar the country from eradicating the infection in the poultry production. The persistence of the hot spatial line of intensity and magnitude over the past 3 years indicates that mobilizing maximum resources on this line to strengthen the current surveillance and control program particularly prior to and in the winter months might be a better means of allocating resources.

## Supporting Information

Text S1 **Formula used for calculation of epidemic doubling time.**
(DOC)
