# Supplementary material for: Spatio-Temporal Magnitude and Direction of Highly Pathogenic Avian Influenza (H5N1) Outbreaks in Bangladesh
Source: PLoS One. 2011 Sep 9;6(9):e24324. doi: 10.1371/journal.pone.0024324 (PMC3170297; doi:10.1371/journal.pone.0024324)
Supplement: Text S1 — Formula used for calculation of epidemic doubling time. (DOC) [file pone.0024324.s001.doc]

Text S1: Formula used for calculation of epidemic doubling time.

The outbreak doubling time from time t_0 to_ t_1_ is:

Here, N_1_ and N_0_ is the respective number of outbreaks at times t_1_ and t_0_.
